# Microbial Primer: *In vivo* biofilm

**DOI:** 10.1099/mic.0.001407

**Published:** 2023-12-05

**Authors:** Kendra P. Rumbaugh, Thomas Bjarnsholt

**Affiliations:** ^1^​ Department of Surgery, Texas Tech University Health Sciences Center, Lubbock, Texas, USA; ^2^​ Department of Immunology and Molecular Microbiology, Texas Tech University Health Sciences Center, Lubbock, Texas, USA; ^3^​ Texas Tech University Health Sciences Center and Burn Center of Research Excellence, Lubbock, Texas, USA; ^4^​ Department of Immunology and Microbiology, Faculty of Health and Medical Science, University of Copenhagen, Copenhagen, Denmark; ^5^​ Department of Clinical Microbiology, Rigshospitalet, Copenhagen, Denmark

**Keywords:** animal model, biofilm, in vivo

## Abstract

In this primer on biofilms and their role in infections, we trace the historical roots of microbial understanding from Van Leeuwenhoek’s observations to Bill Costerton’s groundbreaking work, which solidified biofilms' significance in infections. *In vivo* biofilm research, investigating patient samples and utilizing diverse host models, has yielded invaluable insights into these complex microbial communities. However, it comes with several challenges, particularly regarding replicating biofilm infections accurately in the laboratory. *In vivo* biofilm analyses involve various techniques, revealing biofilm architecture, composition, and behaviour, while gaps in knowledge persist regarding infection initiation and source, diversity, and the Infectious Microenvironment (IME). Ultimately, the study of biofilms in infections remains a dynamic and evolving field poised to transform our approach to combat biofilm-associated diseases.

## Translating the biofilm concept to infection

In the late 1800s, Robert Koch and Louis Pasteur made groundbreaking discoveries regarding the role of microorganisms in infections, with their primary focus being on the isolation and purification of bacteria. They successfully cultivated bacteria using various media, such as potato slices, and solid and liquid media, establishing the foundation for microbiology in both basic science and medical research. Yet, the understanding of microbial behaviour dates back nearly 200 years prior, to Antonie Van Leeuwenhoek’s initial observations, which were of aggregated bacteria. Thus, it has long been recognized that microbes can exist as single cells but also possess the capability to form aggregates, or what we today refer to as biofilms. The extent of aggregation is influenced by factors present in the surrounding environment and microenvironment, as well as the metabolic activity and phenotypic expression of the bacteria.

The significance of biofilms in causing infections became evident when Bill Costerton connected protected, aggregated bacteria on surfaces in Canadian alpine creeks to aggregated bacteria in human infections. These biofilms demonstrated tolerance against antibiotics and the ability to evade host defenses, serving as a reservoir for recurrent infections [1]. Costerton coined the term ‘biofilms’ to describe the bacterial biomaterial covering rocks in alpine creeks, and this term has since been used to refer to aggregated bacteria on various surfaces.

**Fig. 1.**
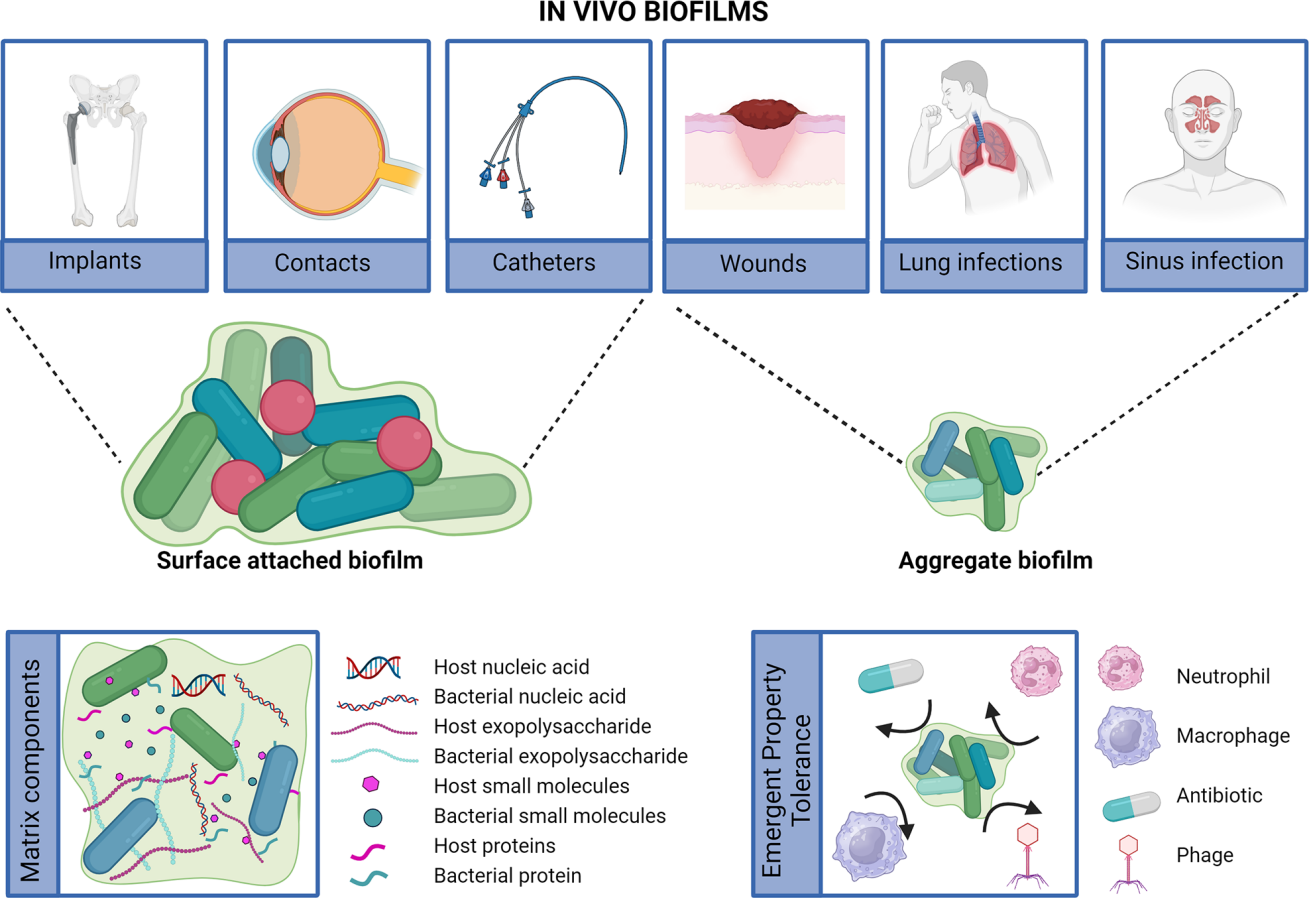
Biofilm in infection. *In vivo* biofilm can be surface attached, as in the case of many foreign-body infections, or present as suspended aggregates, as seen in many tissue-associated infections. Similar to *in vitro* biofilms, biofilms in infection are surrounded by a matrix, but the source of components that make up the matrix is bacterial and host-derived. The major emergent property of *in vivo* biofilm is its exceptional tolerance against killing by factors such as antibiotics and host immune cells. Created with BioRender.com.

Costerton recognized the implications of biofilms in infection causation and strived to raise awareness among microbiologists and clinicians [2]. His mission was to bridge the gap between basic science and medical practice, emphasizing the importance of biofilms in clinical settings. Since then, substantial knowledge has been generated in the field of biofilms. Initially, it was believed that biofilms had to be attached to surfaces and embedded in a self-produced matrix. This perception was influenced by laboratory cultivation methods and the term ‘film’ in biofilm, implying the presence of slime on surfaces. However, it is now widely accepted that suspended aggregates can also qualify as biofilms and that the biofilm matrix includes host components [3] (Fig. 1). Despite differences in appearance and environment, bacteria within biofilms share common phenotypes and characteristics.

## Hallmark characteristics of *in vivo* biofilm

While there are similarities in the phenotypes or qualities of biofilms formed *in vitro* and *in vivo*, there are also distinct differences. One of the most striking disparities lies in the size and structure of *in vivo* biofilms, which are generally much smaller and lack the characteristic architecture seen in biofilms grown *in vitro*. When biofilms develop on the surface of foreign bodies, their size can vary widely, averaging around 1200 µm in diameter [4]. However, biofilms involved in tissue or mucosal infections tend to consist of much smaller aggregates of cells (5–200 µm in diameter) [4]. In both types of *in vivo* biofilms, a mixture of large and small aggregates, along with single cells, can be observed.

Antibiotic tolerance stands out as perhaps the most common and clinically significant emergent property of biofilms [5]. This tolerance is a shared feature of both *in vivo* and *in vitro* biofilms and is primarily attributed to the protective nature of the biofilm matrix and the altered physiology of biofilm-associated cells. The extracellular polymeric substance (EPS) is a hydrated matrix encompassing polysaccharides, proteins, and nucleic acids that envelop biofilm cells, providing structural support and protection. While the EPS of biofilms grown *in vitro* is mostly produced by the microbes within the biofilm, our understanding of the EPS surrounding *in vivo* aggregates is less comprehensive, as well as its role in the antibiotic tolerance characteristic of all biofilms. Nonetheless, it is widely recognized that *in vivo* biofilms integrate matrix materials from the host, such as fibrin, collagen, and/or host nucleic acid (Fig. 1).

Both types of biofilms exhibit spatial and temporal heterogeneity, featuring distinct microenvironments and subpopulations of cells within the biofilm structure. However, biofilms formed *in vivo* may display specific physiological adaptations unique to the host environment. For instance, bacterial biofilms in the human body may express virulence factors or adopt specific metabolic pathways not observed in laboratory-grown biofilms. Similarly, biofilms formed *in vitro* and *in vivo* encounter different nutrient and oxygen conditions. *In vivo* biofilms depend on host-derived nutrients and factors, whereas *in vitro* biofilms are typically cultured in defined media, resulting in distinct nutrient environments. Several other emergent biofilm properties have been recognized and studied *in vitro*, such as architecture, nutrient cycling, exchange of genetic material and quorum sensing [6]. However, very little is understood about these properties *in vivo* or whether they are relevant at all.

It is important to acknowledge that biofilms are highly adaptable and can display significant variation, making it challenging to generalize their phenotypes or qualities. The shared and unique characteristics discussed here represent broad observations that may vary depending on the specific biofilm, host, or experimental conditions.

## Modeling *in vivo* biofilm

Over the past five decades, a multitude of models have been developed to study biofilm-related diseases [7]. Ranging from invertebrate hosts like nematodes and fruit flies to a variety of mammalian hosts, researchers have extensively leveraged these models to explore biofilm-related diseases, the intricate interplay between host and microbes, and the effectiveness of therapeutic interventions.

The choice of a model host is influenced by a variety of factors, including cost, ethical considerations, and technical feasibility. Crucially, it depends on the appropriateness of the host’s anatomy and physiology for the specific disease being investigated. Consequently, for biofilm-related infections relevant to humans, mammals are often regarded as the most suitable hosts. For instance, chinchillas, with their ear anatomy resembling that of humans, have emerged as the gold standard model for otitis media biofilm studies. Similarly, pigs, due to their dermal structure resembling that of humans, are considered ideal for wound biofilm research. Nevertheless, mice are the most frequently used mammalian host due to their cost-effectiveness and the availability of experimental tools.

Broadly categorized, mammalian biofilm models fall into two main types: foreign-body models and tissue infection models. Foreign-body models are designed to simulate specific biofilm infections associated with foreign objects introduced into body cavities or organs. These models can vary widely in terms of the type and location of the foreign object, as well as the method of inoculation and the infecting microbe. Common examples include the subcutaneous, intraperitoneal, or intrauterine introduction of catheters pre-coated with the bacterial strain of interest. Another variation involves implanting a contaminated foreign body into the tibia to replicate conditions akin to osteomyelitis or prosthetic joint infections. Tissue infection models, on the other hand, primarily focus on studying biofilms associated with chronic wound or lung infections. However, they can also simulate biofilm-related conditions in tissues or mucosal surfaces, such as sinusitis, urinary tract infections, or otitis media.

While *in vivo* models offer the advantage of closely mimicking clinical conditions for studying bacteria, they come with several challenges compared to *in vitro* models. Ethical concerns and administrative hurdles, particularly in mammalian live animal experimentation, can be formidable. The costs associated with specific model hosts can be prohibitive, and the variability between individual animals can introduce significant uncertainty. Biological distinctions among animals, species variations, and differences in host responses further complicate the interpretation and reproducibility of experimental outcomes. Moreover, conducting research in mammalian models often demands extensive technical expertise.

In most *in vivo* infection models, an artificially high infection inoculum is necessary to ensure infection. However, this differs starkly from human infections, where only 1–10 % of patients acquire foreign body-related and wound infections and infection likely initiates from small inoculums. Consequently, the aetiology and prerequisites for infection are skewed when using animal models, necessitating careful interpretation of results accordingly.

## Analysing *in vivo* biofilm

Analysing *in vivo* biofilms presents a multifaceted challenge due to the dynamic and complex nature of these microbial communities within living organisms. Nonetheless, many techniques and methodologies to dissect biofilm structure, composition, and behaviour *in vivo* have been established.

Imaging biofilms formed *in vivo* is one of the most common methods of analysis. For real-time monitoring of biofilm infections, bioluminescent bacteria can be engineered to emit light, allowing researchers to track the progression and distribution of the infection within the host. While *in vivo* imaging provides the powerful ability to track infection dynamics over time in live animals, it provides little detail about bacterial spatial distribution or biofilm development.

Conversely, high-resolution imaging methods like confocal laser scanning microscopy (CLSM) and two-photon microscopy allows for visualization of biofilm architecture within the host. While these techniques can provide detailed insights into biofilm thickness, 3D structure, and the distribution of bacterial cells and EPS, imaging biofilms *in vivo* can be demanding. For example, in most *in vivo* scenarios, the biofilm is, in reality, a mixture of small bacterial aggregates and individual cells. These bacterial populations are much smaller in number compared to their *in vitro* counterparts, making their detection and imaging challenging. Coupled with a high degree of bacterial heterogeneity and signal interference from host cells and tissues, the interpretation of experimental findings can be exceedingly complex.

Consequently, most imaging of *in vivo* biofilm is performed *ex vivo* on tissue sections or devices removed from the infected host. To identify specific bacterial species or components within biofilms, fluorescence labelling is frequently employed. Fluorescently tagged antibodies or probes can selectively bind to target molecules, enabling the visualization of bacterial species, host tissues, and matrix components. Additionally, examination of tissue samples through histopathology allows researchers to visualize the impact of biofilm infections on host tissues. When combined with other measurements of the host response (e.g. ELISA for inflammatory markers), this technique helps identify biofilm-related tissue damage, inflammation, and host responses.

In addition to imaging, many *in vivo* biofilm studies include measurements of bacterial load by culture-based methods or quantitative PCR (qPCR). When combined with assessments of overall health of the host, such as body weight, temperature, clinical signs of infection and host immune response, these parameters can provide a reasonable evaluation of infection progression and severity.

To understand the metabolic activity and functional aspects of biofilms *in vivo*, techniques like transcriptomics and proteomics are used to analyse gene expression and protein profiles, providing insights into the biofilm’s adaptability and responses to the host environment. These techniques can also be leveraged to analyse the genetic composition of biofilm communities *in vivo*, identify the presence of specific genes, assess microbial diversity, and detect genetic changes over the course of infection.

Lastly, *in vivo* biofilm models are frequently employed to assess the efficacy of antimicrobial agents, new therapies, and drug delivery systems against biofilm infections. *In vivo* models can be used to determine a drug’s pharmacokinetics, bioavailability, and toxicity profile in addition to its efficacy in eradicating or reducing biofilm-associated infection.

Overall, analysing biofilms *in vivo* is a multidisciplinary endeavour, requiring the integration of various techniques to decipher the intricate interactions between biofilms, host tissues, and the immune response. These approaches collectively contribute to our understanding of biofilm pathogenesis and offer insights into potential strategies for prevention and treatment in clinical settings.

## Gaps in knowledge and avenues for future work

Understanding the limitations and constraints of biofilm model systems is crucial. Equally important is acknowledging what we know and do not know about biofilms within infections. One area that remains poorly understood is the initiation of infections. In all likelihood, infections commence when specific bacteria are present, originating either from the environment or the patient’s own microbiota. These bacteria take advantage of an opportunity, such as a breach in the skin, to establish an infection. This initiation process involves stochastic factors, including the challenge dose and combination of bacterial species, making it challenging to reproduce accurately *in vitro* or in animal models. Furthermore, successful infections depend on the presence of a susceptible host, as the immune system frequently eliminates invading bacteria without causing noticeable infections. Understanding the source of infecting bacteria is essential for a comprehensive grasp of biofilm infection dynamics.

Similar to source of infection, the exact number and phenotype of bacteria needed to sustain chronic infections remains unknown. However, the bacterial phenotype in an inoculum can significantly influence infection outcomes, leading to prolonged lag phases, tolerant subpopulations, and distinct structures. Microscopic evidence suggests that even a small number of bacteria can fuel long-lasting infections and chronic infections progress slowly over weeks to years and typically exhibit focal progression. Bacteria within these infections often display heterogeneous distribution, existing as small aggregates or single cells. These single cells may not resemble fast-growing, susceptible planktonic bacteria found in *in vitro* shaken cultures but can exhibit dormancy and antibiotic tolerance similar to biofilm bacteria. Thus, considering the potential of inoculating infection models with aggregates is vital when studying infections, especially in regard to studying the mechanisms of tolerance and resistance to phagocytosis in biofilm infections.

Bacterial diversity in infections is another area we are only beginning to understand. Infections can be mono- or polymicrobial, depending on stochastic events during the infection process. However, while molecular data like 16S rRNA gene sequencing may suggest multispecies infections, microscopic evidence often indicates limited interaction among these bacteria. Specific anatomical sites are more prone to certain bacterial species, while some infections are predominantly caused by a single species. In some cases, such as mucosal and skin infections, the diversity of infectious bacterial consortia is lower compared to healthy microbiota. In contrast, dermal and respiratory infections tend to be polymicrobial, albeit with varying species composition. It is also evident that each patient and infection can differ significantly in terms of bacterial species and diversity. While fully replicating these complexities in *in vitro* and in animal models is impossible, it is crucial to consider these variations when extrapolating findings to human infections.

Lastly, understanding the microenvironment and the infectious microenvironment (IME) is crucial to future advances. Differences in the microenvironment between *in vitro* and *in vivo* settings play a key role in bacterial persistence and the ability of biofilms exhibit reduced responsiveness to antibiotics. The concept of the IME was inspired by the tumour microenvironment in cancer research and has implications for antibiotic delivery and bacterial distribution within infections [8]. Specifically, alterations in many factors in the IME, such as nutrient availability, oxygen and pH gradients, physical barriers (e.g. cilia) and host matrix components (e.g. collagen and fibrin) can alter bacterial behaviour, create spatial heterogeneity and hinder antibiotic diffusion into the biofilm potentially leading to increased antibiotic resistance and persistence.

## Conclusions

In conclusion, the study of biofilms in the context of infections represents a journey that spans centuries, from the pioneering observations of Antonie Van Leeuwenhoek to the modern understanding of biofilm-associated diseases. The concept of biofilms as a critical factor in infections was solidified by the groundbreaking work of Bill Costerton, who recognized their significance and sought to bridge the gap between basic science and medical practice.

*In vivo* biofilm research has provided invaluable insights into the complexities of microbial communities within living organisms. These models, ranging from invertebrate hosts to mammals, have enabled us to explore biofilm-related diseases, host-microbe interactions, and therapeutic strategies. However, they come with challenges, including ethical considerations, cost, variability, the need for technical expertise and most importantly, the accuracy of the biofilm infection they’re designed to replicate.

Despite significant progress, gaps in our knowledge persist. The initiation of infections, the minimal bacterial load required for chronic infections, the role of bacterial diversity, and the influence of the IME all warrant further investigation. Recognizing these knowledge gaps is essential for advancing our understanding of biofilm-related infections and developing more effective prevention and treatment strategies. Ultimately, the study of biofilms within infections continues to be a dynamic and evolving field with the potential to reshape our approach to combating biofilm-associated diseases.

## References

[1] Marrie TJ, Nelligan J, Costerton JW (1982). A scanning and transmission electron microscopic study of an infected endocardial pacemaker lead. Circulation.

[2] Lappin-Scott H, Burton S, Stoodley P (2014). Revealing a world of biofilms—the pioneering research of bill costerton. Nat Rev Microbiol.

[3] Sauer K, Stoodley P, Goeres DM, Hall-Stoodley L, Burmølle M (2022). The biofilm life cycle: expanding the conceptual model of biofilm formation. Nat Rev Microbiol.

[4] Bjarnsholt T, Alhede M, Alhede M, Eickhardt-Sørensen SR, Moser C (2013). The *in vivo* biofilm. Trends Microbiol.

[5] Ciofu O, Moser C, Jensen PØ, Høiby N (2022). Tolerance and resistance of microbial biofilms. Nat Rev Microbiol.

[6] Bamford NC, MacPhee CE, Stanley-Wall NR (2023). Microbial Primer: an introduction to biofilms - what they are, why they form and their impact on built and natural environments. Microbiology.

[7] Lebeaux D, Chauhan A, Rendueles O, Beloin C (2013). From *in vitro* to *in vivo* models of bacterial biofilm-related infections. Pathogens.

[8] Bjarnsholt T, Whiteley M, Rumbaugh KP, Stewart PS, Jensen PØ (2022). The importance of understanding the infectious microenvironment. Lancet Infect Dis.

